# The Role of Sympathetic Nerves in Osteoporosis: A Narrative Review

**DOI:** 10.3390/biomedicines11010033

**Published:** 2022-12-23

**Authors:** Weifei Zhang, Yuheng Liu, Jixuan Xu, Chen Fan, Bin Zhang, Pin Feng, Yu Wang, Qingquan Kong

**Affiliations:** 1 Department of Orthopedics, Orthopedic Research Institute, West China Hospital, Sichuan University, Chengdu 610041, China; 2 Hospital of Chengdu Office of People’s Government of Tibetan Autonomous Region (Hospital C. T.), Sichuan University, Chengdu 610041, China; 3 Department of Orthopedics, Hospital of Chengdu Office of People’s Government of Tibetan Autonomous Region (Hospital C. T.), Orthopedic Research Institute, West China Hospital, Sichuan University, Chengdu 610041, China

**Keywords:** sympathetic nerves, osteoporosis

## Abstract

Osteoporosis, a systemic bone disease, is characterized by decreased bone density due to various reasons, destructed bone microstructure, and increased bone fragility. The incidence of osteoporosis is very high among the elderly, and patients with osteoporosis are prone to suffer from spine fractures and hip fractures, which cause great harm to patients. Meanwhile, osteoporosis is mainly treated with anti-osteoporosis drugs that have side effects. Therefore, the development of new treatment modalities has a significant clinical impact. Sympathetic nerves play an important role in various physiological activities and the regulation of osteoporosis as well. Therefore, the role of sympathetic nerves in osteoporosis was reviewed, aiming to provide information for future targeting of sympathetic nerves in osteoporosis.

## 1. Introduction

Osteoporosis, a systemic bone disease, is characterized by decreased bone density due to various reasons, destructed bone microstructure, and increased bone fragility [1], and it is defined as the density of the femoral neck or lumbar spine that is less than −2.5 measured by QCT (quantitative computed tomography) or dual energy X-ray. If the bone density is between −2.5 and −1.5, it is called low bone mass [2,3,4]. The incidence of osteoporosis is high, and about 9% of Americans over the age of 50 and 200 million women worldwide develop osteoporosis [5]. The most serious common complication of osteoporosis is fracture. In western societies, one in three women and one in five men suffer osteoporotic fractures for the rest of their lives after the age of 50, especially spine and hip fractures that increase the mortality of osteoporotic patients [6]. The current treatment of osteoporosis is mainly through drug therapy, including antiresorptive agents and drugs that promote bone formation, for example: bisphosphonates estrogen modulators, teriparatide, denosumab, etc. A Rankl inhibitor, denosumab, is a drug recently approved by the FDA to treat osteoporosis. The usage is to inject under the skin every 6 months, which has good anti-osteoporosis curative effects. While these treatments are accompanied with serious adverse events such as thromboembolism, increased risk of stroke, and hypocalcemia, some drugs, such as zoledronic acid, require hospitalization to check renal function, which is inconvenient for clinical use [7,8,9,10,11,12,13,14], and thus it is necessary to develop new treatment methods.

Sympathetic and parasympathetic nerves make up the autonomic nerves, and the autonomic nerve controls vital physiological functions such as heart beat, respiration, digestion, blood pressure, metabolism, etc. [15,16,17,18,19]. The activities of the sympathetic nervous system are relatively extensive; stimulating the sympathetic nerves can cause vasoconstriction of abdominal viscera and skin peripheral blood vessels, strengthening and acceleration of the heartbeat, mydriasis, decreased secretion of digestive glands, increased working ability of tired muscles, etc., The activity of sympathetic nerves is to mainly guarantee the physiological needs of the human body in states of stress [20,21,22,23,24]. Sympathetic nerves also play a regulatory role in osteoporosis [25]. The occurrence of osteoporosis is mainly caused by the decrease of osteogenesis and osteoclastosis [26,27,28,29,30,31]. We mainly discuss five aspects: the structural basis of sympathetic regulation of osteoporosis, the phenomenon of sympathetic nerve regulation of osteoporosis, sympathetic nerve regulation of osteogenesis, sympathetic nerve regulation of osteoclastogenesis, and drugs for treating osteoporosis through regulating sympathetic nerve activity, aiming to provide information for the treatment of osteoporosis.

## 2. Methods

**Figure 1:**
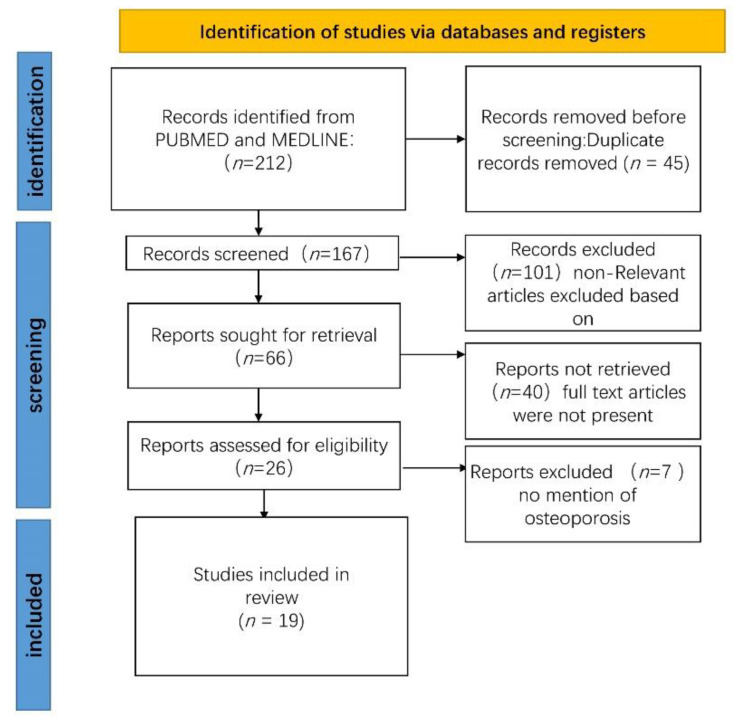
PRISMA flowchart showing the process of study selection.

We used “sympathetic nerve” and “osteoporosis”, “sympathetic nerve” and “osteoblast “, “sympathetic nerve” and “osteoclast“ as keywords, and searched all the articles from 1953 to 2022 in PUBMED and MEDLINE. We included only publications published in English and selected those findings that were, in our opinion, the most important. We further analyzed these articles; we mainly selected papers from the past 5 years but also included well-respected older publications (Figure 1). The registration number is: INPLASY2022120079. 

## 3. Results and Discussion

### 3.1. Structural Basis of Sympathetic Regulation of Osteoporosis

Sympathetic neurons are located in the lateral horn of the thoracolumbar segment of the spinal cord, and their fibers originate from the corresponding spinal segment and terminate in the paravertebral ganglia or prevertebral ganglia, called preganglionic fibers [32,33,34]. After leaving the spinal cord, preganglionic fibers may ascend or descend for several segments in the sympathetic chain and then terminate in ganglia. A preganglionic fiber often has many branches that are connected with different postganglionic neurons, thus resulting in a “scattered” excitation effect [35,36,37].

There are two types of neurotransmitters released by sympathetic nerves: the neurotransmitter released by all sympathetic preganglionic nerve endings is acetylcholine, and all sympathetic postganglionic nerve endings release norepinephrine [38,39,40].

The receptors of the sympathetic nerve are adrenergic receptors, which are divided into two categories, namely α and β [41,42]. α receptors are divided into α1 and α2: α1 is distributed on the presynaptic membrane and vascular smooth muscle, which mainly causes vasoconstriction when excited; α2 is mainly distributed on the presynaptic membrane of noradrenergic nerves and produces negative feedback regulation and inhibition on the secretion of NE when excited [43,44,45]. β receptors are divided into β1, β2, and β3; β1 is mainly distributed on cardiomyocytes. After the activation of β1 receptors, it has positive effects on the myocardium, thus resulting in a series of reactions of myocardial excitation; β2 receptors are mainly distributed on smooth muscles, such as blood vessels, smooth muscle, alimentary canal smooth muscle, bronchial smooth muscle, etc., and this receptor can cause smooth muscle relaxation after activation; β3 receptors are mainly distributed in white and brown adipose tissue to regulate energy metabolism [46,47,48,49].

In fact, many studies have confirmed the presence of sympathetic receptors on the cell membranes of osteoblasts and osteoclasts, which provides a structural basis for sympathetic regulation of the progression of osteoporosis [50,51,52,53,54,55].

### 3.2. The Phenomenon of Sympathetic Nerve Regulation of Osteoporosis

The discovery of many phenomena indicates that the dysregulation of sympathetic nerves is related to the occurrence of osteoporosis. Interestingly, the regulation of sympathetic nerves in osteoporosis is complex, and sympathetic nerve dysfunction leads to osteoporosis. Inhibition of sympathetic nerves can also treat osteoporosis, which seems contradictory. The specific mechanism is currently unclear, and it requires more and more systematic research.

Many scholars have observed that sympathetic nerve dysfunction leads to osteoporosis: Iris A Enríquez-Pérez et al. found that diabetes-induced reduction in sympathetic nerve fiber density was associated with femoral neck bone loss in mice [56]; Sharareh Roshanzamir et al. discovered that sympathetic nerve disorder caused by electrical burn was associated with osteoporosis [57]; C J Stephens et al. reported a case of reflex sympathetic dystrophy in a patient with severe osteoporosis [58]. The findings of these studies proved that sympathetic nerve disorders indeed lead to the occurrence of osteoporosis, but there is no relevant report on the mechanism at present.

However, according to other scholars, inhibiting sympathetic nerve activity can increase bone mass and prevent osteoporosis from progressing. Based on T Sato et al. the selective β2-adrenergic antagonist butoxamine reduces bone loss in sympathetically hyperactive osteoporotic rats [59]. Wenping Zhang et al. revealed that the beta-blocker propranolol could improve bone mass and inhibit osteoporosis progression in osteoporotic rats [60].

### 3.3. Sympathetic Regulation of Osteogenesis

The main reason for the occurrence of osteoporosis is the decrease in osteogenesis and osteoclastosis [61,62,63,64,65,66,67,68]. Osteoblast differentiation is a central step in bone formation [26,27,28,29,30,31]. Many studies have illustrated that the sympathetic nerve affects bone formation and the progression of osteoporosis by regulating osteoblast differentiation (Table 1).

At present, most scholars hold the view that sympathetic nerve activation inhibits osteoblast differentiation and osteogenesis. In addition, it promotes the formation of osteoporosis. Ziyan Wang et al. indicated that long-term use of adrenergic drugs increases the odds of fracture, and the β2-adrenergic receptor agonist terbutaline directly inhibits osteogenesis by impairing osteogenic differentiation and mineralization [69]. Yupeng Wu et al. confirmed that the inhibition of sympathetic nerve activity can promote the osteogenic differentiation capacity of osteoblasts and mesenchymal stem cells [70]. Takayuki Yamada et al., discovered that the activation of the sympathetic nerve inhibits osteoblast differentiation [71]. Yoon Jung Choi et al. revealed that the α-blocker doxazosin activates ERK1/2 in stem cells to promote osteogenic differentiation [72]. The view that the sympathetic nerve inhibits osteogenesis is also supported by other researchers [52,73].

However, some scholars agree that the activation of sympathetic nerves promotes osteogenesis and inhibits the progression of osteoporosis. On the basis of Yun Ma et al. a reduction in norepinephrine leads to a reduction in osteogenesis [74]. Ji-Ye He et al. revealed that sympathetic neurons can promote osteoblast differentiation through the BMP signaling pathway [75]. Takuya Uemura et al. found that epinephrine accelerates osteoblastic differentiation by enhancing bone morphogenetic protein signaling through a cAMP/protein kinase A signaling pathway [76].

All in all, there is a certain degree of controversy about the role of sympathetic nerves in osteogenesis. At present, most researchers argue that sympathetic nerve activation promotes osteogenesis, and some scholars hold the opposite view, which requires additional systematic research in the future.

### 3.4. Sympathetic Nerve Regulation of Osteoclastogenesis

The process of bone resorption is as follows: monocytes differentiate into osteoclasts that attach to the old bone area, secrete acidic substances to dissolve minerals, and secrete protease substances to digest old bones [77,78,79,80,81,82]. It is well known that an important cause of osteoporosis is increased osteoclastic activity [83,84,85,86]. The opinion of most scholars is that increased sympathetic nerve activity promotes the process of osteoclastic bone formation (Table 2).

Florent Elefteriou et al. found that leptin activates sympathetic nerves to increase osteoclast differentiation, leading to increased bone resorption and eventually leading to the progression of osteoporosis [87]; H Cao et al. found that the sympathetic nervous system increases the RANKL/OPG ratio of peripheral blood mononuclear cells through β-2 adrenergic receptors, promotes their differentiation into osteoclasts, and increases bone resorption [88]; Sarah J Aitken et al. found that the activation of sympathetic nerves promotes osteoclastogenesis and bone resorption [89]; U Frediani et al. found that catecholamines stimulate osteoclast differentiation by binding to β2 adrenergic receptors, thus promoting the progression of osteoporosis [90]. Most importantly, other researchers have made similar findings [54,91].

In general, the role of sympathetic nerves in osteoclasts is relatively well established, and sympathetic nerve activation promotes the process of osteoclast differentiation.

### 3.5. Drugs for Treating Osteoporosis through Regulating Sympathetic Nerve Activity

The most common treatment for osteoporosis is anti-osteoporosis drugs, such as bisphosphonates, raloxifene, estrogens, parathyroid hormone, etc. These drugs can achieve certain clinical effects, but there are also clinical limitations and side effects [92,93,94,95,96,97]. For example, zoledronic acid requires testing of renal function before being infused during an outpatient clinic visit, and the use of zoledronic acid may have side effects on the jaw [98,99,100,101]; raloxifene may cause venous thromboembolism [102,103,104]; estrogen therapy for osteoporosis leads to an increased risk of gynecological tumors [105,106,107]; the parathyroid hormone analog teriparatide may cause lower blood pressure in osteoporosis patients [108,109,110]. Therefore, it is of clinical significance to develop new drugs for the treatment of osteoporosis. Given our previous discussion, sympathetic nerves play an important role in the progression of osteoporosis, and many potential drugs have been found to inhibit osteoporosis through sympathetic nerves (Table 3).

Hao Chen et al. found that the β2-adrenergic antagonist propranolol has a therapeutic effect on osteoporosis [111]; T Sato et al., discovered that the selective beta2-adrenergic antagonist butoxamine ameliorates osteoporosis with an overactive sympathetic nervous system [59]; Hideo Shimizu et al. proved that cilnidipine inhibits the progression of osteoporosis in ovariectomized hypertensive rats by inhibiting sympathetic nerve activity [112]; Wenping Zhang et al. revealed that the beta-blocker propranolol increases bone mass in osteoporotic mice [60].

In general, scholars agree that drugs that inhibit sympathetic nerve activity have potential effects on the treatment of osteoporosis. For example, propranolol is a potential drug for osteoporosis treatmen;, propranolol is a drug mainly used to treat isocardial arrhythmias of atrial and ventricular premature beats and is also effective for hypertension. If a patient with arrhythmia or high blood pressure happens to suffer from osteoporosis, then propranolol may be a very suitable drug, which requires comprehensive clinical research; certainly, propranolol should not be used in patients with bronchospasm and cardiogenic shock [113].

## 4. Conclusions

Osteoporosis is a common disease, and the resulting spine and hip fractures are detrimental to the life quality and safety of the elderly. The current treatment for osteoporosis usually involves anti-osteoporosis drugs, but these drugs have side effects. It is of clinical importance to find new anti-osteoporosis drugs. 

Sympathetic nerves are part of the autonomic nervous system and have a wide range of regulatory effects. Osteoporosis is mainly caused by decreased osteogenesis and increased osteoclasts. There are sympathetic receptors on their cell membranes, and sympathetic neuromodulation of osteoporosis provides a structural basis. Sympathetic inactivation may lead to osteoporosis, and the inhibition of sympathetic activity also inhibits osteoporosis progression, which seems paradoxical and requires more research. Many scholars argue that sympathetic nerves can inhibit osteogenesis, but some scholars hold the opposite view. Sympathetic nerve-promoting osteoclasts are recognized by many researchers. Furthermore, some drugs can inhibit the progression of osteoporosis by regulating sympathetic nerve activity, such as propranolol, which may become a therapeutic drug for patients with arrhythmia or high blood pressure who happen to suffer from osteoporosis in the future.

## Figures and Tables

**Table 1:** Sympathetic regulation of osteogenesis.

Scholars	Views	Research Model	Mechanisms	References
Ziyan Wang et al.	inhibit osteogenesis	mice	β2-adrenergic receptor agonist	[69]
Yupeng Wu et al.	inhibit osteogenesis	rabbits	β-receptor antagonist	[70]
Takayuki Yamada et al.	inhibit osteogenesis	cell	β2-adrenergic receptor agonist	[71]
Yoon Jung Choi et al.	inhibit osteogenesis	cell	α receptor antagonist	[72]
Yun Ma et al.	inhibit osteogenesis	mice	β2-adrenergic receptor agonist	[52]
Haifang Li et al.	inhibit osteogenesis	cell	β2 and β3 adrenergic receptor agonist	[73]
Yun Ma et al.	promote osteogenesis	mice	norepinephrine	[74]
Ji-Ye He et al.	promote osteogenesis	cell	bone morphogenetic protein	[75]
Takuya Uemura et al.	promote osteogenesis	cell	epinephrine	[76]

**Table 2:** Sympathetic regulation of osteoclasts.

Scholars	Views	Research Model	Mechanisms	References
Florent Elefteriou et al.	promote osteoclast	mice	β2-adrenergic receptor agonist	[87]
H Cao et al.	promote osteoclast	rats	β2-adrenergic receptor agonist	[88]
Sarah J Aitken et al.	promote osteoclast	cell	β2-adrenergic receptor agonist	[89]
U Frediani et al.	promote osteoclast	cell	β2-adrenergic receptor agonist	[90]
T Takeuchi et al.	promote osteoclast	cell	β-receptor agonist	[91]
Yuka Okada et al.	promote osteoclast	rats	β-receptor agonist	[54]

**Table 3:** Potential drugs inhibit osteoporosis through sympathetic nerves.

Potential Drugs	Scholars	Research Model	Mechanisms	References
propranolol	Hao Chen et al.	mice	β2-adrenergic antagonist	[111]
butoxamine	T Sato et al.	rats	selective beta2-adrenergic antagonist	[59]
cilnidipine	Hideo Shimizu et al.	rats	inhibiting sympathetic nerve activity	[112]
propranolol	Wenping Zhang et al.	rats	beta-blocker	[60]

## Data Availability

Not applicable.
